# DASH diet as a preventive strategy for sarcopenia: integrated observational and mendelian randomization analyses in US adults

**DOI:** 10.1186/s12986-025-01021-z

**Published:** 2025-10-23

**Authors:** Jiayi Zheng, Xiaofeng Chen, Ruihan Peng, Bo Li, Yiting Lin, Xianbin Cai

**Affiliations:** 1 Department of Gastroenterology, The First Affiliated Hospital of Shantou University Medical College, Shantou, 515041 Guangdong China; 2 Shenzhen University Affiliated South China, Shenzhen, 518000 Guangdong China

**Keywords:** Dietary patterns, DASH diet, Sarcopenia, Diet intervention, NHANES, Mendelian randomization

## Abstract

**Background:**

Despite the potential of dietary patterns as modifiable interventions, most studies focus on single nutrients and the epidemiological evidence on combined dietary patterns and sarcopenia remains inconclusive.

**Objective:**

Based on NHANES data from 2011 to 2018, we systematically assessed the dose-response relationship between five dietary indices (DASHI, MEDI, AHEI, DII, HEI-2020) and sarcopenia, aiming to provide an evidence-based basis for the development of nutritional intervention strategies.

**Methods:**

Dietary indices were calculated based on 24-hour dietary questionnaire recall and sarcopenia was assessed by ASMBMI. Weighted multivariate logistic regression models adjusted for sociodemographic, lifestyle, and clinical covariates were applied. Restricted cubic splines were used to test for non-linear relationships. The potential causal relationship between dietary components and sarcopenia was further analyzed using MR.

**Results:**

Among 6,210 participants (491 with sarcopenia), higher DASHI scores demonstrated a dose-response protective effect. In the fully adjusted model, the DASHI continuous score was negatively associated with the risk of sarcopenia (OR = 0.83, 95% CI: 0.72–0.95, *P* = 0.008). Quartile trend tests were significant (*P* for trend = 0.018), with a 50% reduction in risk in the highest quartile (Q4) compared to Q1 (OR = 0.50, 95% CI: 0.30–0.84, *P* = 0.011). With regard to dietary components, macronutrients (energy, carbohydrates, proteins, fats), B vitamins, vitamin D, calcium and magnesium were found to be protective against sarcopenia (*P* < 0.05). Causal associations between multiple dietary components and features associated with sarcopenia by MR analysis were consistent with the DASH diet structure.

**Conclusion:**

The DASH diet demonstrates a robust inverse association with sarcopenia, likely mediated by its anti-inflammatory properties and nutrient synergy. These findings prioritize DASH-based interventions for high-risk populations. It may help reduce the healthcare burden imposed by sarcopenia.

**Statement of significance:**

This study is pioneering in its use of nationally representative data to systematically compare the effects of multiple dietary indices on sarcopenia, providing a high-quality, evidence-based foundation for the development of prevention guidelines based on overall dietary composition.

## Introduction

Sarcopenia is an age-related, generalized degenerative disease of skeletal muscle, manifested by progressive loss of muscle mass and strength, which often occurs in association with a variety of chronic diseases and can lead to adverse health outcomes such as falls, fractures and frailty [1].Sarcopenia was officially included in the International Classification of Diseases (ICD-10-CM) in 2016 [2]. According to the 2018 revised criteria of the European Working Group on Sarcopenia in the Elderly, the diagnosis of sarcopenia must meet the criteria of reduced muscle strength, reduced muscle mass, or both [3]. Sarcopenia is a costly public health problem, with studies suggesting that hospitalization costs for sarcopenic patients were five times higher than those for non-sarcopenic patients and that the total annual cost of hospitalization was $40.4 billion [4, 5]. Epidemiological studies have shown that the global prevalence of sarcopenia is approximately 13% and that it is significantly and positively associated with the risk of cardiovascular disease and chronic kidney disease [6, 7]. The development of sarcopenia is associated with multiple mechanisms such as protein metabolism disorders, chronic inflammation, mitochondrial dysfunction, etc., in which nutritional interventions are of central importance as modifiable factors.

Currently, there is no effective drug treatment program and clinical management relies mainly on healthy lifestyle interventions, including a science-based diet and regular exercise [8]. Skeletal muscle metabolism depends on exogenous nutritional support, of which high-quality protein, rich in essential amino acids and vitamin D play a key role in maintaining muscle homeostasis [9]. Studies have confirmed that inadequate protein intake and vitamin D deficiency are common nutritional problems in patients with sarcopenia [10, 11]. Existing nutritional intervention studies have mostly focused on single nutrients or food components, neglecting the synergistic or antagonistic effects among dietary components. In fact, high-quality dietary patterns based on dietary indices can significantly improve muscle mass and function [12]. Such dietary patterns are usually plant-based, i.e. using plants as the main source of protein, and also include the intake of large amounts of fruits and vegetables, nuts and seeds, sources of high-quality fats such as olive oil and vegetable oils, and limiting the consumption of red meat and processed foods. These dietary patterns have anti-inflammatory and antioxidant properties that help prevent metabolic syndrome [13–15]. Although existing studies suggest an association between dietary patterns and muscle health, the strength of the evidence needs to be improved and is limited by small regional sample studies [16].

Using the National Health and Nutrition Examination Survey (NHANES) database, the present study systematically explored the mechanisms of association between various dietary indices and sarcopenia, analyzed the impact of dietary patterns on the development of sarcopenia, and provided an evidence-based basis for the development of nutritional intervention strategies. Mendelian randomization supplemented causal inference of multiple dietary components associated with dietary indices and traits associated with sarcopenia that could not be obtained from observational study.

## Materials and methods

### Survey description

This study was based on data from the 2011–2018 US National Health and Nutrition Examination Survey (NHANES), and detailed information about the study design is publicly available (https://www.cdc.gov/nchs/) [17]. NHANES is a nationally representative cross-sectional survey that includes a wide range of health and nutrition data, including detailed dietary records, clinical examinations, and laboratory test results. Demographic, anthropometric, dietary, and disease questionnaires from NHANES were used for this analysis. Written informed consent was obtained from NHANES participants, and the study protocol was approved by the Research Ethics Review Board of the National Center for Health Statistics and the Human Use Review Board of the U.S. Army Research Institute of Environmental Medicine.

### Study population

**Fig. 1:**
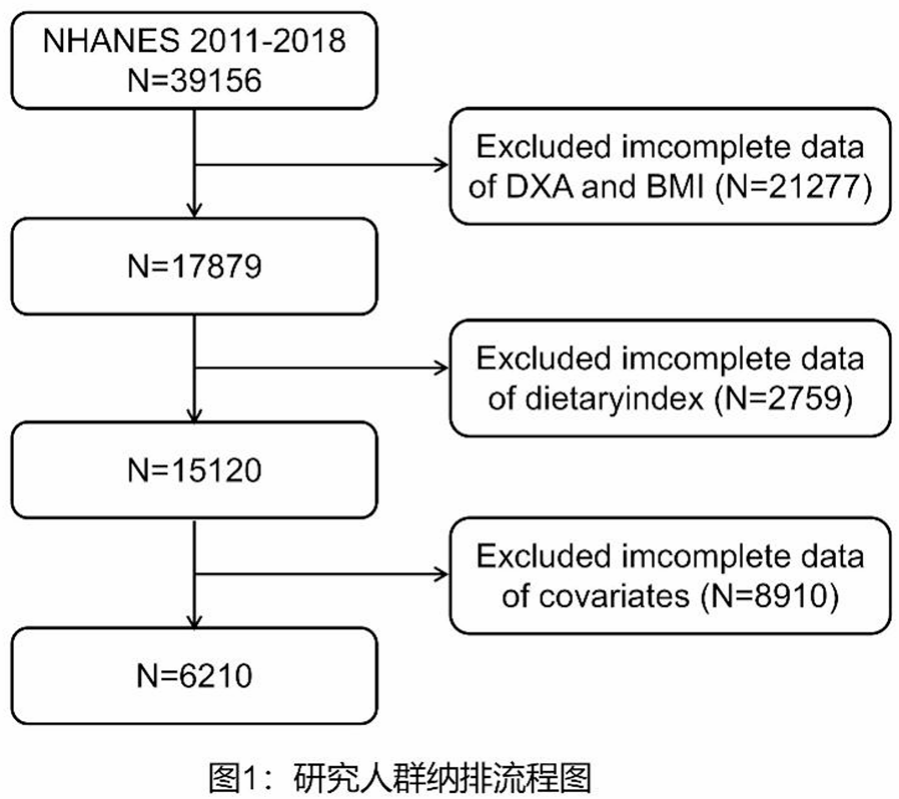
Population inclusion and exclusion flowchart

The data used in this study were from NHANES 2011–2018 because they contain dietary questionnaire data that can be used to calculate dietary metrics, dual-energy X-ray absorptiometry (DXA) measurements, and BMI to assess sarcopenia. A total of 39,156 participants were enrolled in these cycles, of which 17,879 were included because of complete DXA and BMI information. A further 11,669 participants were excluded because they did not meet the inclusion criteria: (i) missing information on dietary intake for dietary indices calculation (*n* = 2759); (ii) incomplete information on other covariates (*n* = 8910). The final analysis included 6210 participants (flowchart in Fig. 1), with a total of 491 participants diagnosed with sarcopenia.

### Sarcopenia

In accordance with the guidelines established by the Foundation for the National Institutes of Health (FNIH), the Appendicular Skeletal Muscle Mass Adjusted for Body Mass Index (ASMBMI) is a method of assessing skeletal muscle mass, utilizing the ratio of Appendicular Skeletal Muscle Mass (ASM) to body mass index (BMI). The assessment of skeletal muscle mass is determined by the ASMBMI, with sarcopenia defined as < 0.512 m^2^ in women and < 0.789 m^2^ in men. Dual-energy X-ray absorptiometry (DXA) is utilized to assess muscle mass of the arms and legs, facilitating the calculation of limb skeletal muscle mass. Individuals with weight > 136 kg, height > 196 cm, and BMI ≤ 12 or ≥ 60 kg/m^2^ were excluded from the calculation.

### Assessment of dietary indices

The collection of dietary information was achieved through the utilization of 24-hour dietary recalls on two consecutive days [18], with subsequent calculation in accordance with the guidelines stipulated by the USDA Diet Study Food and Nutrition Database. The calculation of dietary indices was conducted employing a standardized algorithm, with the calculation of five dietary indices (DASHI, MEDI, AHEI, DII, and HEI2020) being derived from the dietary intake data. The corresponding algorithms and R-codes of the dietaryindex package can be accessed at https://github.com/jamesjiadazhan/. The dietaryindex package is a flexible and validated tool that enables the standardized calculation of dietary indices in epidemiological and clinical studies [19, 20]. The calculation process consists of two steps: firstly, portion sizes are determined for each food and nutritional category, and then individual dietary indices are calculated. Higher dietary scores represent greater adherence to an individual’s healthy dietary pattern.

The DASH diet promotes increased intake of fruits, vegetables, whole grains, low-fat dairy products, nuts, and legumes, while limiting sodium, red and processed meats, and sugary beverages [21, 22]. DASHI is a scoring tool used to quantify the extent to which an individual’s dietary pattern meets the recommended criteria of the DASH diet. In essence, it determines whether a diet meets the recommended goals of the DASH diet by assessing the intake of food groups or nutrients. A higher score indicates that the diet meets the health goals of the DASH diet. The Mediterranean diet, a dietary pattern characterized by an abundance of plant foods, olive oil, fish and nuts, is primarily utilized for the primary prevention of chronic diseases [14]. MEDI is typically calculated based on the frequency of food intake or the amount of food consumed. AHEI serves as an indicator that assesses the quality of the diet based on the amount of food consumed in a healthy eating pattern. It takes into account the intake of foods and nutrients such as vegetables, fruits, whole grains, nuts, fish, red and processed meats, sugar-sweetened beverages, saturated fats, trans fats, sodium, etc [23]. Calculation of the AHEI emphasizes the variety and quality of foods. The calculation is scored based on the difference between the recommended intake of each food component and the actual intake of the individual. DII is an indicator specifically designed to assess the inflammatory potential of the diet. It takes into account the intake of nutrients such as vitamin A, vitamin B6, vitamin C, vitamin E, folate, magnesium, zinc, selenium, saturated fats, monounsaturated fats, polyunsaturated fats, cholesterol, dietary fiber, caffeine, alcohol and others [24]. HEI2020 is used to assess the consistency of an individual’s diet with the US Dietary Guidelines. It encompasses the intake of various foodstuffs and nutrients, including total fruits, whole fruits, total vegetables, dark green and legume vegetables, total protein, seafood and plant proteins, whole grains, dairy products, fatty acid ratios, refined grains, sodium, added sugars, saturated fats, and more [25].

### Covariates

We collected covariates associated with constipation and carotenoids intake through literature review [26, 27]. These covariates included age, gender, race (Mexican American, Non-Hispanic black, Non-Hispanic white, and Other races), marital status (Married or Unmarried), education levels (Below high school, HS/GED, Some college, College), poverty-income ratio (PIR < 1 or ≥ 1), body mass index (BMI), physical activity (a minimum of 150 min of moderate to high-intensity physical activity per week) [28], smoking, drinking, diabetes, cardiovascular disease (CVD), hypertension, energy intake and protein intake. BMI was grouped as underweight (< 18.5 kg/m^2^) or normal weight (≥ 18.5 and < 25 kg/m^2^), overweight (≥ 25 and < 30 kg/m^2^), and obese (≥ 30 kg/m^2^). Classification of smoking was as follows: Never: smoked under 100 cigarettes in their lifetime; Former: smoked over 100 cigarettes in their lifetime and smoke not at all now; and Now: smoked over 100 cigarettes in their lifetime and smoke some or all of the time. Excessive alcohol consumption was defined as daily alcohol intake exceeding 20 g for men and 10 g for women. Participants were diagnosed with hypertension if they met any of the following criteria: (i) self-reported diagnosis by a medical professional; (ii) used anti-hypertensive medication; or (iii) average systolic blood pressure of at least 130 mmHg or average diastolic blood pressure of at least 80 mmHg. The diagnostic criteria for diabetes were: (i) doctor told; or (ii) glycohemoglobin HbA1c ≥ 6.5%; or (iii) use of diabetes medication or insulin. CVD (congestive heart failure, coronary heart disease, angina pectoris, heart attack and stroke) was determined by self-reported questionnaires. Energy and protein intakes were obtained from the 24-hour dietary questionnaire recalls.

### Statistical analysis

In this study, the sample weights provided by NHANES will be used for statistical analyses to ensure that the results are representative of the U.S. population. The normality of variable distributions was assessed using histogram distribution analysis, Q-Q plots, and the Kolmogorov-Smirnov test. Continuous variables that followed a normal distribution were expressed as mean ± standard deviation (SD), while skewed continuous variables were reported as median (interquartile range [IQR]). Categorical variables were summarized as frequencies and percentages (%). Comparisons of continuous variables across groups were conducted using either the independent samples Student’s t-test or the Mann-Whitney U-test, depending on the normality of the data distribution. Categorical data were analyzed using the chi-square test as appropriate.

We used weighted multifactor regression analyses to assess the association between dietary indices and sarcopenia. Dietary indices were included in the analyses as continuous and four-categorical variables. To further explore the possible mechanisms of the effect of dietary patterns on sarcopenia, we also analyzed the association of detailed dietary components with sarcopenia. We constructed 3 models: Model 1 was not adjusted for any covariates. Model 2 was adjusted for age, gender, race, BMI, PIR, marital status, education levels, physical activity, smoking, drinking. Model 3 additionally adjusted for diabetes, CVD, hypertension, energy intake and protein intake. In addition, mediation analyses were used to investigate whether the association between DASHI and sarcopenia could be explained by multiple dietary components after adjusting for covariates in Model 3. We conducted restricted cubic spline model to develop smooth curves to examine the possible nonlinear dose-response associations between dietary scores and sarcopenia. Finally, subgroup analyses stratified by age, sex, race, education levels, diabetes, CVD, and hypertension were performed. Considering serum vitamin D as a potential covariate, multifactorial regression models during sensitivity analyses were further adjusted for serum 25(OH)D levels to fully explore the stability of the association. The calculated effect sizes and *P* values from all these models were reported and compared. All analyses were performed using R Statistical Software (Version 4.2.2, http://www.R-project.org, The R Foundation) and Free Statistics analysis platform (Version 2.1, Beijing, China, http://www.clinicalscientists.cn/freestatistics). A two-sided *P* value < 0.05 was considered statistically significant.

### MR design

The detailed description of the overall MR study design and assumptions is shown in **supplementary materials**. A properly constructed MR is based on three assumptions: (i) genetic variants are linked to risk factors; (ii) genetic variants are not influenced by confounding factors; and (iii) genetic variants impact outcomes solely through risk factors [29].

### MR analysis

In this MR analysis, we used instrumental variables to individually examine the relationship between potential diet-related risk factors and traits associated with sarcopenia. Exposure factors encompassed multiple dietary components, including fruits, vegetables, whole grains, red meat, fish, nuts and legumes, milk, beverages and juices, coffee, tea, fatty acids, ultra-processed foods, vitamin and mineral supplements, and never eat eggs, dairy, wheat, sugar [30]. We chose appendicular lean mass (BIA), handgrip strength (left), handgrip strength (right), whole-body and limbs fat-free mass, physical activity in the last 4 weeks, and usual walking pace as the phenotypic traits relevant to sarcopenia [31]. Single nucleotide polymorphism (SNP) data associated with the exposure factors were obtained from the GWAS Catalog database, which included information on SNP loci, effect sizes, standard errors, and p-values for each exposure factor, and data on the phenotypic traits relevant to sarcopenia were also extracted from the SNPs that were matched by the instrumental variables to the exposure data. A two-sample Mendelian randomization method was used and analyses were performed using the TwoSampleMR package. The correlation assumption was met by selecting SNPs of genome-wide significance (*P* < 5 × 10 − 8) as instrumental variables. Exposure data were formatted and instrumental variables were screened by chain imbalance correction (clump_r2 = 0.001). Instrumental variables with an F-statistic less than 10 were excluded to minimize potential weak instrument bias. Causal relationships between exposure factors and outcomes were assessed using various mendelian randomization methods, including inverse variance weighted (IVW), weighted median (WM), and MR-Egger regression, and sensitivity analyses were performed by heterogeneity test, horizontal pleiotropy test (*P* value for MR-Egger intercept < 0.05 was considered as the potential presence of horizontal pleiotropy), MR-PRESSO analysis, and leave-one-out analysis. The results of the analyses were presented through visualization tools such as scatter plots, forest plots and funnel plots to present a more intuitive picture of the potential causal relationship between exposure factors and sarcopenia.

## Results

### Baseline characteristics of participants

As demonstrated in Table 1, the baseline characteristics of participants have been categorized according to the presence or absence of sarcopenia. With regard to the investigation of sarcopenia, a total of 6210 participants were included from 2011 to 2018, of which 491 were diagnosed with sarcopenia. The mean age of the participants was 39.3 ± 11.5 years, with 48.0% male and 52% female subjects. The mean age of the sarcopenia population was 43.6 ± 11.3 years, with a higher proportion of females (56.4%). The mean DASHI, MEDI, AHEI, DII and HEI2020 scores for the entire included participants were 3.7 ± 1.3, 3.6 ± 1.1, 38.0 ± 11.7, 1.2 (−0.2, 2.3), and 50.9 ± 12.1, respectively. Compared to the reference population, patients with sarcopenia had a higher prevalence of diabetes, CVD, and hypertension, with mean energy, protein, carbohydrate, sugar, fiber and fat intake relatively lower, as were serum 25(OH)D levels, and both ASM and ASMBMI were relatively low, suggesting insufficient muscle mass in line with the diagnostic criteria for sarcopenia.

**Table 1:** Baseline characteristics of participants with and without sarcopenia

Characteristics	Total (*n* = 6210)	Without sarcopenia (n = 5719)	Sarcopenia (n = 491)	*P* value
Age (years)	39.3 ± 11.5	39.0 ± 11.4	43.6 ± 11.3	< 0.001
Gender (%)				0.04
Male	2983 (48.0)	2769 (48.4)	214 (43.6)	
Female	3227 (52.0)	2950 (51.6)	277 (56.4)	
Race (%)				< 0.001
Mexican American	880 (14.2)	701 (12.3)	179 (36.5)	
Non-Hispanic Black	1296 (20.9)	1271 (22.2)	25 (5.1)	
Non-Hispanic White	2365 (38.1)	2222 (38.9)	143 (29.1)	
Other	1669 (26.9)	1525 (26.7)	144 (29.3)	
Education levels (%)				< 0.001
Below high school	1022 (16.5)	880 (15.4)	142 (28.9)	
college	1857 (29.9)	1770 (30.9)	87 (17.7)	
HS/GED	1316 (21.2)	1192 (20.8)	124 (25.3)	
Some college	2015 (32.4)	1877 (32.8)	138 (28.1)	
Marital status (%)				0.022
No	2451 (39.5)	2281 (39.9)	170 (34.6)	
Yes	3759 (60.5)	3438 (60.1)	321 (65.4)	
PIR (%)				< 0.001
Not poor	4826 (77.7)	4474 (78.2)	352 (71.7)	
Poor	1384 (22.3)	1245 (21.8)	139 (28.3)	
BMI (%)				< 0.001
Normal weight	1878 (30.2)	1838 (32.1)	40 (8.1)	
Obese	2252 (36.3)	1915 (33.5)	337 (68.6)	
Overweight	1983 (31.9)	1870 (32.7)	113 (23)	
Underweight	97 (1.6)	96 (1.7)	1 (0.2)	
Smoking (%)				< 0.001
Current smoker	1376 (22.2)	1301 (22.7)	75 (15.3)	
Former	1058 (17.0)	969 (16.9)	89 (18.1)	
Never	3776 (60.8)	3449 (60.3)	327 (66.6)	
Drinking (%)				0.001
No	5039 (81.1)	4614 (80.7)	425 (86.6)	
Yes	1171 (18.9)	1105 (19.3)	66 (13.4)	
Activity (%)				< 0.001
No	3799 (61.2)	3441 (60.2)	358 (72.9)	
Yes	2411 (38.8)	2278 (39.8)	133 (27.1)	
Diabetes (%)				< 0.001
No	5568 (89.7)	5186 (90.7)	382 (77.8)	
Yes	642 (10.3)	533 (9.3)	109 (22.2)	
CVD (%)				< 0.001
No	5996 (96.6)	5542 (96.9)	454 (92.5)	
Yes	214 (3.4)	177 (3.1)	37 (7.5)	
Hypertension (%)				< 0.001
No	3738 (60.2)	3507 (61.3)	231 (47)	
Yes	2472 (39.8)	2212 (38.7)	260 (53)	
Kcal(kcal/d)	2143.0 ± 836.9	2162.4 ± 845.8	1917.1 ± 686.2	< 0.001
Protein(g/d)	84.8 ± 37.0	85.4 ± 37.5	77.4 ± 30.8	< 0.001
Carbohydrate(g/d)	259.0 ± 108.5	261.1 ± 109.8	235.4 ± 89.0	< 0.001
Sugar(g/d)	111.5 ± 66.2	112.7 ± 66.9	98.1 ± 55.2	< 0.001
Fiber(g/d)	17.5 ± 9.7	17.6 ± 9.8	16.8 ± 8.3	0.102
Fat(g/d)	81.2 ± 38.4	82.0 ± 38.8	71.8 ± 31.8	< 0.001
Vitamin D (mcg/d)	3.4 (1.7, 5.9)	3.4 (1.7, 6.0)	3.1 (1.6, 5.3)	0.031
25(OH)D (nmol/L)	60.6 ± 25.0	60.9 ± 25.2	56.9 ± 22.2	< 0.001
ASM (kg)	22.6 ± 6.1	22.9 ± 6.1	19.7 ± 5.4	< 0.001
ASMBMI (m^2^)	0.8 ± 0.2	0.8 ± 0.2	0.6 ± 0.1	< 0.001
DASHI	3.7 ± 1.3	3.7 ± 1.3	3.9 ± 1.3	0.017
MEDI	3.6 ± 1.1	3.6 ± 1.1	3.5 ± 1.0	0.337
AHEI	38.0 ± 11.7	38.1 ± 11.8	36.5 ± 10.3	0.003
DII	1.2 (−0.2, 2.3)	1.1 (−0.3, 2.3)	1.5 (0.3, 2.6)	< 0.001
HEI2020	50.9 ± 12.1	50.9 ± 12.1	50.0 ± 11.7	0.118
DASHI, n (%)				0.235
Q1(0.48,2.78)	1553 (25.0)	1444 (25.2)	109 (22.2)	
Q2(2.78,3.58)	1552 (25.0)	1437 (25.1)	115 (23.4)	
Q3(3.58,4.57)	1552 (25.0)	1418 (24.8)	134 (27.3)	
Q4(4.57,8.50)	1553 (25.0)	1420 (24.8)	133 (27.1)	
MEDI, n (%)				0.27
Q1(0,3.0)	1375 (22.1)	1275 (22.3)	100 (20.4)	
Q2(3.0,3.5)	1093 (17.6)	992 (17.3)	101 (20.6)	
Q3(3.5,4.5)	2117 (34.1)	1948 (34.1)	169 (34.4)	
Q4(4.5,8.5)	1625 (26.2)	1504 (26.3)	121 (24.6)	
AHEI, n (%)				0.012
Q1(7.84,29.24)	1553 (25.0)	1419 (24.8)	134 (27.3)	
Q2(29.24,37.05)	1552 (25.0)	1410 (24.7)	142 (28.9)	
Q3(37.05,45.77)	1552 (25.0)	1433 (25.1)	119 (24.2)	
Q4(45.77,81.64)	1553 (25.0)	1457 (25.5)	96 (19.6)	
DII, n (%)				< 0.001
Q1(−4.86,0.21)	1553 (25.0)	1473 (25.8)	80 (16.3)	
Q2(−0.21,1.17)	1552 (25.0)	1423 (24.9)	129 (26.3)	
Q3(1.17,2.32)	1552 (25.0)	1417 (24.8)	135 (27.5)	
Q4(2.32,4.74)	1553 (25.0)	1406 (24.6)	147 (29.9)	
HEI2020, n (%)				0.491
Q1(10.00,41.88)	1553 (25.0)	1420 (24.8)	133 (27.1)	
Q2(41.88,49.82)	1552 (25.0)	1429 (25)	123 (25.1)	
Q3(49.82,58.92)	1552 (25.0)	1427 (25)	125 (25.5)	
Q4(58.92,91.80)	1553 (25.0)	1443 (25.2)	110 (22.4)	

### The association between dietary indices and sarcopenia

**Fig. 2:**
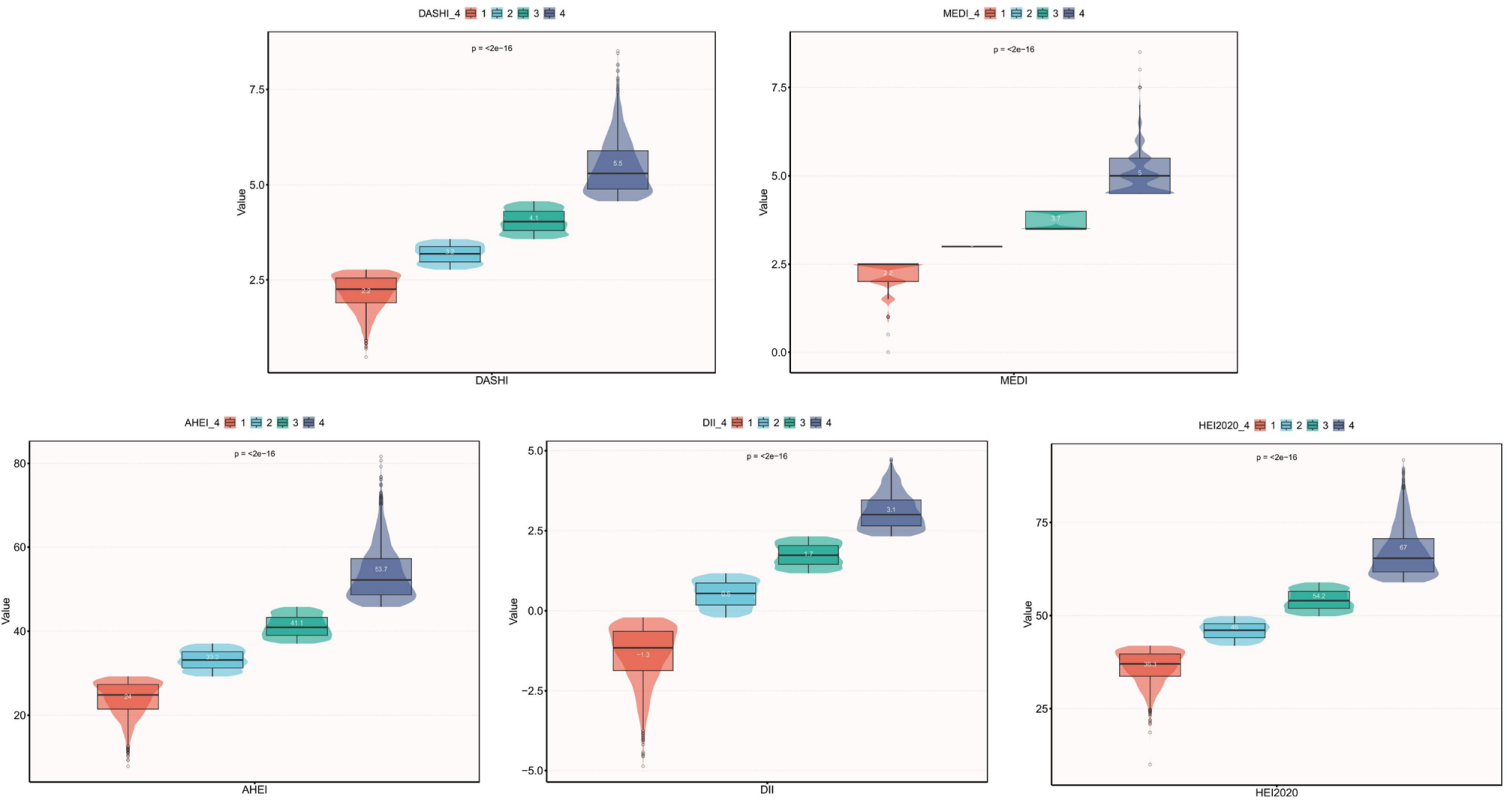
Visualization of the IQR cutoffs for dietary indices

The weighted multifactor regression analyses were shown in Table 2. In unadjusted Model 1, all dietary indices except DASHI were significantly associated with sarcopenia, with MEDI, AHEI, and HEI2020 being negatively associated with the prevalence of sarcopenia and DII being positively associated. These associations changed statistically after adjusting for relevant covariates and dividing all dietary scores into quartiles. In the fully adjusted model (Model 3), the DASHI continuous score was negatively associated with the risk of prevalence of sarcopenia (OR = 0.83, 95% CI: 0.72–0.95, *P* = 0.008). Quartile trend tests were significant (*P* for trend = 0.018), with a 50% reduction in risk in the highest quartile (Q4) compared to Q1 (OR = 0.50, 95% CI: 0.30–0.84, *P* = 0.011). AHEI (OR = 0.99, *P* = 0.044), with HEI2020 (OR = 0.99, *P* = 0.049) showed protective trends in continuous analyses, but quartile trends were not significant after fully adjusting for covariates (*P* > 0.05). MEDI in continuous analyses after fully adjusting for covariates (OR = 0.88, *P* = 0.097) and interquartile trends (*P* = 0.097) were not significant. The pro-inflammatory diet assessed by DII in Model 1 (OR = 1.26, 95% CI: 1.14–1.38, *P* < 0.001) and Model 2 (OR = 1.27, 95% CI: 1.12–1.44, *P* < 0.001) was positively associated with sarcopenia, but the association was attenuated after adjusting for chronic disease, energy and protein intake (OR = 1.14, 95% CI: 0.99–1.30, *P* = 0.064) Fig. 2.

**Table 2:** Relations between dietary indices and sarcopenia; OR, odds ratio; CI, confidence interval; DASHI, dietary approaches to stop hypertension index; MEDI, mediterranean diet index; AHEI, alternate healthy eating Index; DII, dietary inflammatory Index; HEI-2020, healthy eating Index-2020

	Model 1			Model 2			Model 3		
	OR	95% CI	*P* value	OR	95% CI	*P* value	OR	95% CI	*P* value
DASHI									
Continuous	0.88	0.77, 1.01	0.061	0.89	0.77, 1.02	0.092	0.83	0.72, 0.95	**0.008**
Quartile			0.103*			0.148*			0.018*
Q1(0.48,2.78)	Reference			Reference			Reference		
Q2(2.78,3.58)	0.83	0.59, 1.18	0.288	0.86	0.59, 1.25	0.413	0.81	0.57, 1.17	0.256
Q3(3.58,4.57)	0.93	0.58, 1.48	0.751	0.89	0.56, 1.41	0.598	0.80	0.52, 1.23	0.292
Q4(4.57,8.50)	0.61	0.37, 0.98	**0.043**	0.64	0.38, 1.08	0.091	0.50	0.30, 0.84	**0.011**
MEDI									
Continuous	0.83	0.74, 0.93	**0.002**	0.90	0.78, 1.03	0.128	0.88	0.76, 1.02	0.097
Quartile			0.052*			0.363*			0.272*
Q1(0,3.0)	Reference			Reference			Reference		
Q2(3.0,3.5)	1.10	0.69, 1.74	0.689	1.10	0.68, 1.77	0.684	1.04	0.64, 1.67	0.878
Q3(3.5,4.5)	0.90	0.57, 1.43	0.661	0.94	0.56, 1.56	0.789	0.86	0.52, 1.44	0.555
Q4(4.5,8.5)	0.64	0.40, 1.05	0.074	0.80	0.46, 1.38	0.405	0.77	0.46, 1.32	0.330
AHEI									
Continuous	0.98	0.96, 0.99	**< 0.001**	0.98	0.97, 0.99	**0.009**	0.99	0.97, 1.00	**0.044**
Quartile			0.002*			0.030*			0.094*
Q1(7.84,29.24)	Reference			Reference			Reference		
Q2(29.24,37.05)	0.89	0.69, 1.16	0.394	0.85	0.61, 1.19	0.340	0.87	0.62, 1.23	0.417
Q3(37.05,45.77)	0.81	0.55, 1.18	0.258	0.73	0.49, 1.09	0.122	0.81	0.54, 1.20	0.273
Q4(45.77,81.64)	0.47	0.30, 0.74	**0.002**	0.57	0.33, 0.97	**0.039**	0.66	0.40, 1.10	0.103
DII									
Continuous	1.26	1.14, 1.38	**< 0.001**	1.27	1.12, 1.44	**< 0.001**	1.14	0.99, 1.30	0.064
Quartile			< 0.001*			0.001*			0.106*
Q1(−4.86,0.21)	Reference			Reference			Reference		
Q2(−0.21,1.17)	1.54	0.95, 2.48	0.076	1.51	0.90, 2.52	0.114	1.26	0.73, 2.17	0.396
Q3(1.17,2.32)	2.18	1.30, 3.66	**0.004**	2.20	1.25, 3.86	**0.008**	1.58	0.88, 2.82	0.119
Q4(2.32,4.74)	2.59	1.53, 4.39	**< 0.001**	2.68	1.42, 5.07	**0.004**	1.64	0.80, 3.33	0.165
HEI2020									
Continuous	0.98	0.97, 0.99	**< 0.001**	0.99	0.97, 1.00	**0.034**	0.99	0.97, 1.00	**0.049**
Quartile			0.002*			0.073*			0.096*
Q1(10.00,41.88)	Reference			Reference			Reference		
Q2(41.88,49.82)	0.85	0.63, 1.15	0.286	0.93	0.65, 1.32	0.656	0.92	0.66, 1.29	0.618
Q3(49.82,58.92)	0.72	0.51, 1.03	0.070	0.77	0.53, 1.12	0.168	0.82	0.56, 1.20	0.300
Q4(58.92,91.80)	0.54	0.37, 0.79	**0.002**	0.67	0.41, 1.09	0.100	0.68	0.43, 1.09	0.108

Model 1: no covariates were adjusted. Model 2: adjusted for age, gender, race, marital status, education levels, poverty-income ratio (PIR), body mass index (BMI), physical activity, smoking and drinking. Model 3: adjusted for age, gender, race, marital status, education levels, poverty-income ratio (PIR), body mass index (BMI), physical activity, smoking, drinking, diabetes, CVD, hypertension, energy intake and protein intake

*P for trends. Bold values indicate statistical significance (*P* value < 0.05)

### The association between dietary components and sarcopenia

The weighted multivariate regression analyses of dietary components and sarcopenia risk in the NHANES database revealed significant associations across three models Table 3. After fully adjusting for covariates, the macronutrients: energy, carbohydrate, fiber and fat had a protective effect against sarcopenia (OR < 1, *P* < 0.05). Protein, a core substrate of muscle metabolism, significantly reduced the risk of sarcopenia by 12% per 10 g/d increment (OR = 0.88, 95% CI: 0.84–0.93, *P* < 0.001). B vitamins, especially vitamin B2 (OR = 0.60, 95% CI: 0.49–0.74, *P* < 0.001) had the most significant protective effect against sarcopenia. Vitamin D (OR = 0.93, 95% CI: 0.89–0.97, *P* = 0.001) reduced the risk by 7% per 1 mcg/d increment. Magnesium was associated with a 36% reduction in incremental risk per 100 mg/d (OR = 0.64, 95% CI: 0.54–0.75, *P* < 0.001).

**Table 3:** Relations between dietary components and sarcopenia; OR, odds ratio; CI, confidence interval; model 1: no covariates were adjusted. model 2: adjusted for age, gender, race, marital status, education levels, poverty-income ratio (PIR), body mass index (BMI), physical activity, smoking and drinking abuse. model 3: adjusted for age, gender, race, marital status, education levels, poverty-income ratio (PIR), body mass index (BMI), physical activity, smoking, drinking abuse, diabetes, CVD and hypertension

	Mode 1			Model 2			Model 3		
	OR	95% CI	*P* value	OR	95% CI	*P* value	OR	95% CI	*P* value
Energy (100 kcal/d)	0.96	0.94, 0.98	< 0.001	0.95	0.93, 0.98	< 0.001	0.95	0.93, 0.98	< 0.001
Protein (10 g/d)	0.91	0.88, 0.95	< 0.001	0.88	0.84, 0.93	< 0.001	0.88	0.84, 0.93	< 0.001
Carbohydrate (100 g/d)	0.79	0.70, 0.90	< 0.001	0.75	0.63, 0.89	0.002	0.76	0.64, 0.90	0.003
Sugar (100 g/d)	0.70	0.58, 0.85	< 0.001	0.66	0.50, 0.87	0.004	0.67	0.51, 0.88	0.006
Fiber (g/d)	0.97	0.95, 0.99	0.001	0.96	0.94, 0.98	< 0.001	0.96	0.94, 0.98	< 0.001
Fat (10 g/d)	0.94	0.89, 0.98	0.009	0.92	0.87, 0.97	0.005	0.92	0.87, 0.97	0.005
Saturated fatty acids (10 g/d)	0.86	0.75, 0.99	0.034	0.82	0.71, 0.95	0.012	0.82	0.70, 0.95	0.011
Vitamin B1 (mg/d)	0.72	0.59, 0.88	0.002	0.71	0.55, 0.92	0.012	0.71	0.55, 0.92	0.011
Vitamin B2 (mg/d)	0.65	0.55, 0.77	< 0.001	0.60	0.49, 0.75	< 0.001	0.60	0.49, 0.74	< 0.001
Vitamin B6 (mg/d)	0.71	0.60, 0.83	< 0.001	0.70	0.59, 0.85	< 0.001	0.71	0.59, 0.85	< 0.001
Vitamin B12 (mcg/d)	0.95	0.91, 0.98	0.007	0.95	0.90, 0.99	0.025	0.95	0.90, 0.99	0.031
Vitamin C (10 mg/d)	0.97	0.94, 0.99	0.004	0.97	0.95, 0.99	0.012	0.97	0.95, 0.99	0.019
Vitamin D (mcg/d)	0.93	0.90, 0.97	< 0.001	0.93	0.89, 0.97	0.001	0.93	0.89, 0.97	0.001
Vitamin K (10 mcg/d)	0.96	0.93, 0.98	< 0.001	0.96	0.94, 0.99	0.006	0.96	0.94, 0.99	0.007
Calcium (100 mg/d)	0.91	0.89, 0.94	< 0.001	0.90	0.86, 0.93	< 0.001	0.90	0.86, 0.93	< 0.001
Phosphorus (100 mg/d)	0.93	0.91, 0.95	< 0.001	0.91	0.88, 0.94	< 0.001	0.91	0.88, 0.94	< 0.001
Magnesium (100 mg/d)	0.67	0.59, 0.77	< 0.001	0.63	0.54, 0.75	< 0.001	0.64	0.54, 0.75	< 0.001
Iron (10 mg/d)	0.76	0.60, 0.95	0.018	0.74	0.56, 0.98	0.038	0.74	0.56, 0.99	0.043
Sodium (100 mg/d)	0.98	0.97, 0.99	< 0.001	0.98	0.97, 0.99	< 0.001	0.98	0.97, 0.99	< 0.001

### Analysis of the role of dietary components in the correlation between DASHI and sarcopenia using the mediation effect model

**Fig. 3:**
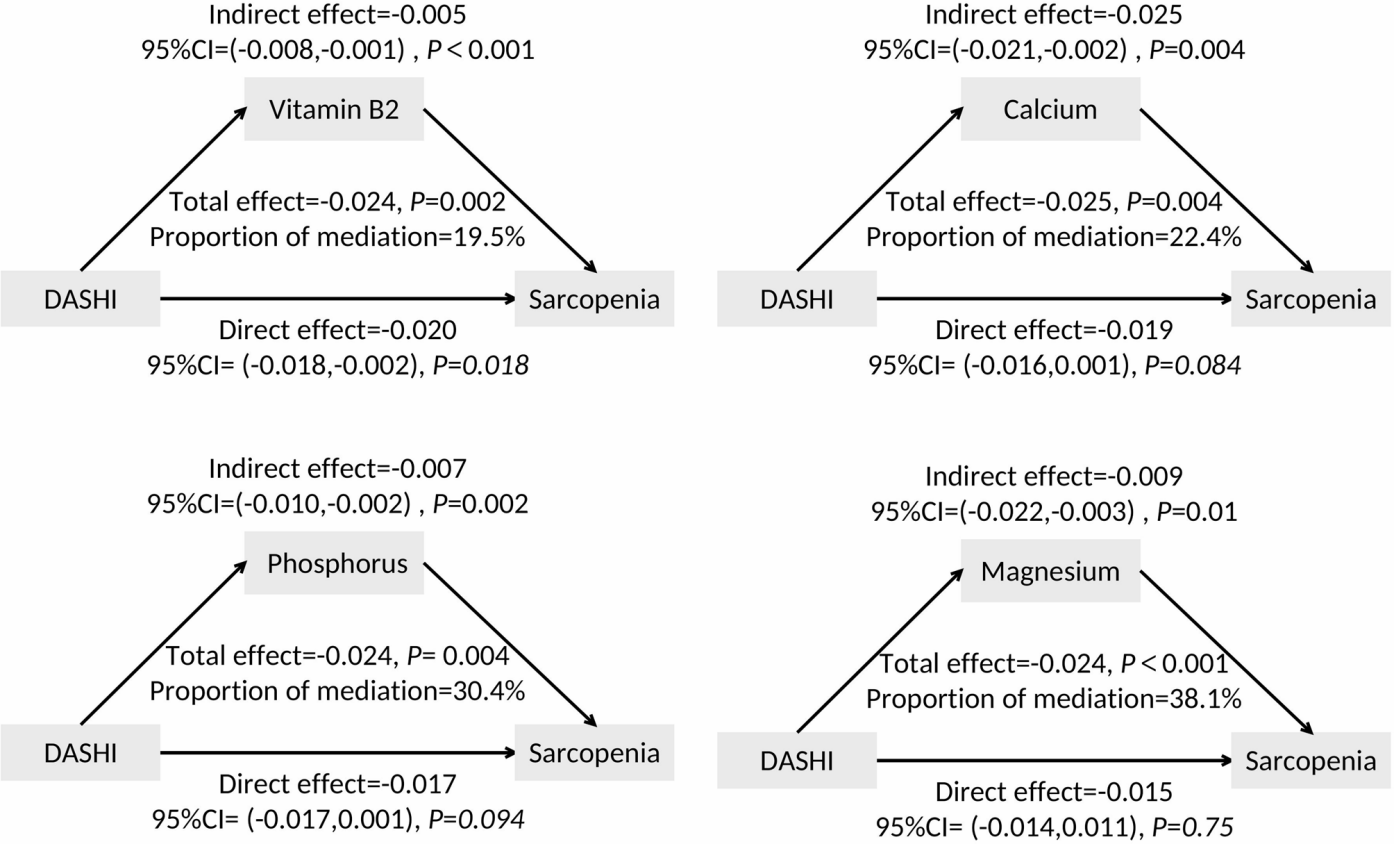
Mediation analysis of the association between DASH and sarcopenia by vitamin B2, calcium, phosphorus and magnesium

Mediation analysis showed that vitamin B2, calcium, phosphorus and magnesium mediated the association between DASHI and sarcopenia Fig. 3. The proportions of indirect effects mediated by vitamin B2, calcium, phosphorus and magnesium were 19.5%, 22.4%, 30.4% and 38.1%. Magnesium mediated the most significant proportion of indirect effects in the association between DASHI and sarcopenia.

### The linear relationship between dietary indices and sarcopenia

**Fig. 4:**
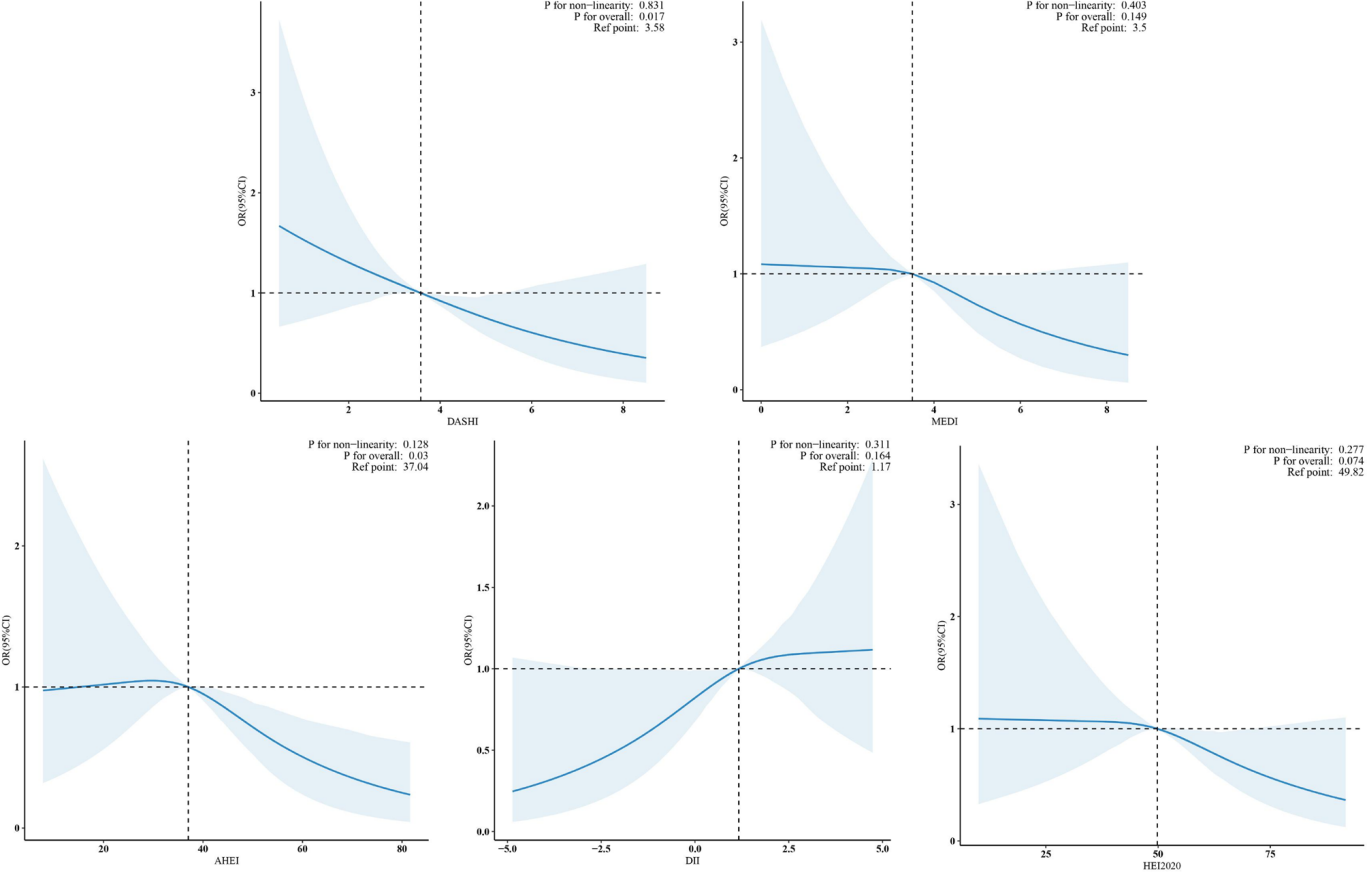
Visualization of the relationship between DASHI, MEDI, AHEI, DII and HEI2020 and sarcopenia, with the solid blue line indicating a smooth curve fit between the variables and the blue area indicating the 95% confidence interval of the fit

Smoothed curve fitting results Fig. 4 also showed that DASHI (*P*-overall: 0.017, *P*-nonlinear: 0.831), MEDI (*P*-overall: 0.403, *P*-nonlinear: 0.149), AHEI (*P*-overall: 0. 030, *P*-nonlinear: 0.128), DII (*P*-overall: 0.164, *P*-nonlinear: 0.311) and HEI2020 (*P*-overall: 0.074, *P*-nonlinear: 0.277) did not show a nonlinear association with sarcopenia.

### Sensitivity analyses and subgroup analyses

**Fig. 5:**
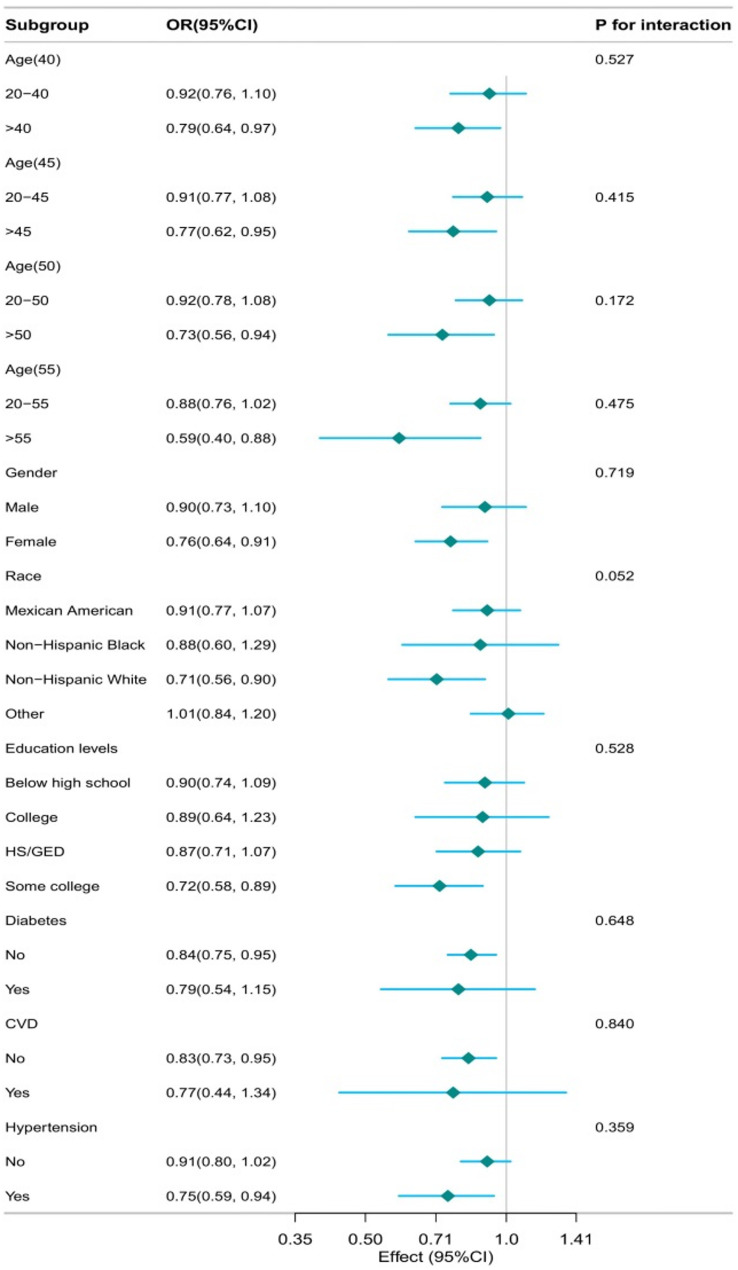
Subgroup analyses and interactions tests

Given the role of serum vitamin D as an important covariate, after further adjustment for serum 25(OH)D on the basis of Model 3, the association between DASHI and sarcopenia remained negative after continuous analysis (OR = 0.84, 95% CI: 0.73–0.96, *P* = 0.014) and interquartile grouping (*P* for trend = 0.026) remained negatively correlated after and there was no significant change in the magnitude of the effect size. To assess whether the association between DASH and sarcopenia was influenced by potential covariates, we performed subgroup analyses and interaction tests stratified by age (grouped by cutoff points of 40, 45, 50, and 55), sex, race, education levels, diabetes, CVD, and hypertension Fig. 5. The association between DASH and sarcopenia was nearly identical across subgroups and there was no significant effect of the association between DASH and sarcopenia in all subgroups (*P* > 0.05 for all interactions).

### MR result

**Fig. 6:**
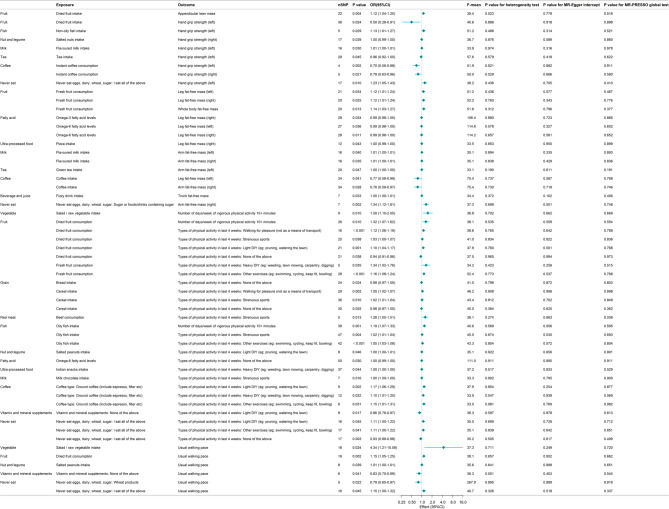
Mendelian random estimation of the association between sarcopenia-related traits and dietary components genetically predicted by IVW method

In this study, we identified dietary components with potential causal associations with sarcopenia characteristics by MR Fig. 6. Positive causal associations were found between vegetables, fruits, grains, fish, milk, and no sugar consumption and sarcopenia-related traits, while negative causal associations were found between coffee, tea, fatty acids, and ultra-processed foods and sarcopenia-related traits. The associations between these dietary components and sarcopenia-related traits were consistent with the recommended intake and strict intake of dietary components of the DASH diet. All relevant findings from the MR analysis can be found in Supplementary materials, including the five analysis methods used in the MR analysis.

## Discussion

The present study, which analyzed the results of the survey of US adult population, found that the DASH dietary pattern was negatively associated with the risk of developing sarcopenia, with higher scores inversely associated with lower risk. This association potentially involved DASH dietary compositional characteristics. Among these dietary components, vitamin B2, calcium, phosphorus and magnesium mediated the association between the DASH dietary pattern and sarcopenia. The results of MR analyses of multiple dietary components and sarcopenia-related traits supported the positive impact of the DASH diet on sarcopenia prevention. Pro-inflammatory diets were associated with sarcopenia, but this association was attenuated after adjusting for chronic diseases, energy and protein intake, suggesting that these factors may exist on a causal pathway, but that potential confounders cannot be excluded. The protective effects of the traditional Mediterranean diet (MED) and Healthy Eating Index (AHEI, HEI2020) protective effects were attenuated after adjusting for nutrient metabolic factors, suggesting that the mechanisms may involve mediating pathways such as protein intake.

The DASH diet is associated with pattern linked to blood pressure regulation and cardiovascular health [32]. Although sodium and potassium intake have been shown to be protective against sarcopenia, excessive intake may increase the risk of diseases such as hypertension, heart diseases, and chronic kidney disease [33], and is therefore recommended in moderation for patients with sarcopenia. Whole grains are rich in magnesium, which regulates muscle protein synthesis by modulating the activation of the mTOR signaling pathway, and maintains mitochondrial energy metabolism to prevent protein senescence, so moderate magnesium intake could contribute to prevent sarcopenia in the diet of middle-aged and elderly adults [34, 35]. Inadequate vitamin D and calcium intake have been shown to be associated with an elevated risk of sarcopenia [36]. Calcium, a key element in muscle contraction, works in concert with vitamin D to regulate muscle energy metabolism and myosin homeostasis, which in turn affects skeletal muscle mass and function [37].The high dairy intake recommended by the DASH diet provides vitamin D and vitamin B12, which are protective of muscle health [38]. B vitamins, especially vitamin B2, have significant protective effects against sarcopenia. In our study, vitamin B2 showed a strong protective effect and indirectly mediated the association between the DASH diet and sarcopenia, which is consistent with previous studies [39]. Vitamin B2 exerts an antioxidant function and participates in muscle energy metabolism through its conversion to flavin adenine dinucleotide (FAD) and flavin mononucleotide (FMN). Furthermore, vitamin B2 indirectly affects muscle protein synthesis and repair by activating vitamin B6 [40–42]. Therefore, an adequate intake of vitamin B2 is essential for maintaining muscle strength and endurance. Branched-chain amino acids (leucine, isoleucine and valine) in dairy products regulate myosin metabolism in both directions through the mTOR pathway: promoting synthesis and inhibiting catabolism [43].The DASH diet contains proteins derived mainly from dairy products and plants, and these high-quality proteins are easily digested and absorbed [44]. Studies have demonstrated that the high-quality protein intake included in the DASH diet maintains muscle strength while reducing fat mass [45]. The independent effects of specific anti-inflammatory components of the DASH diet (e.g., magnesium, vitamin C) have been validated by multifactorial regression analysis and Mendelian randomization analysis. The anti-inflammatory properties of the DASH diet have been demonstrated by mechanisms such as a reduction in high-sensitivity CRP, a positive improvement in leukocyte subpopulations, and leukopenia [46–48]. Reduced levels of inflammatory factors, including TNF-α, IL-6 and IGF-1, have been associated with sarcopenia [49], but the anti-inflammatory mechanisms by which the DASH diet modulates protein synthesis still need to be elucidated in depth.

The positive association between DII and sarcopenia found in the present study was partially the same as in previous studies [50], except that the positive association between DII and sarcopenia was attenuated after adjusting for chronic disease, energy, and protein intakes, complementing the fact that pro-inflammatory diets may affect sarcopenia through multiple mechanisms of co-morbidities, inflammatory damage, and indirect nutritional deficiencies. In addition, the Mediterranean diet was not independently associated with sarcopenia [51], a phenomenon that may stem from geographical differences in dietary culture [45], highlighting the need for cross-cultural validation. Previous cohort studies have shown no significant association between dietary patterns and sarcopenia [52, 53], which may be influenced by recall bias and adherence bias, and need to be validated by more standardized assessment methods. The present study supports the use of the DASH diet as a preferred dietary strategy for sarcopenia prevention especially for people at high risk of metabolic syndrome. Given the prevalence of inadequate dietary intake in patients with sarcopenia, it is recommended that health screening for people at risk of sarcopenia focus on inadequate protein intake, B vitamins, calcium, magnesium, and inflammatory markers. Clinical interventions could focus on the potential benefits of combining high-quality protein and anti-inflammatory diets.

This study has the following strengths: it is the first systematic use of NHANES data to comprehensively study the association of multiple dietary indices with sarcopenia. While previous studies have focused on the effects of single dietary components or nutrients on sarcopenia, we used comprehensive dietary pattern scores to provide more rigorous analyses of the overall dietary structure, which provide a more practical guide to developing dietary patterns for the prevention and management of sarcopenia. The results are more robust by adjusting covariates multidimensionally and further validating the effect of serum vitamin D in sensitivity analyses. Mendelian randomization supplemented causal associations that could not be inferred from cross-sectional study and enhanced the strength of evidence. However, this study also has limitations: The population involved in this study was aged 20–59 years, which was inconsistent with the typical sarcopenia risk group (> 60 years). Therefore, there is a need to expand future studies in older populations. Additionally, our study was based on U.S. data. Whether these findings can be widely applied in other regions remains to be seen. Although numerous covariates were adjusted for, dietary assessment via 24-hour recalls was subject to inherent variability. It should be particularly noted that DASHI was constructed using only two days of dietary data, which may introduce measurement error and result in regression dilution bias. Such bias would attenuate the true exposure-outcome associations in continuous models. Consequently, caution was warranted when interpreting causal relationships. As the study was based on a cross-sectional survey and lacked long-term follow-up data, reverse causality may have existed despite the use of Mendelian randomization to verify causal associations. Despite the use of sensitivity analyses and subgroup analyses, it was not possible to completely exclude the influence of other potential covariates.

## Conclusions

This study confirms that the DASH dietary pattern reduces the risk of sarcopenia by comprehensively assessing multiple dietary indices, and that the protective effect may be realized through the synergistic and anti-inflammatory effects of each nutrient. It breaks through the single-nutrient assessment of sarcopenia risk and provides evidence-based support for the primary prevention of sarcopenia based on an integrated dietary pattern. Future randomized controlled trials or long-term cohort studies are still needed to clarify the specific role of different dietary patterns in the etiology and management of sarcopenia.

## Data Availability

No datasets were generated or analysed during the current study.

## Abbreviations

AHEI: Alternate healthy eating index
ASMBMI: Appendicular skeletal muscle mass adjusted for body mass index
DASHI: Dietary approaches to stop hypertension index
DII: Dietary inflammatory index
HEI-2020: Healthy eating index-2020
MEDI: Mediterranean diet index
MR: Mendelian randomization
NHANES: National health and nutrition examination survey
